# A prospective seroepidemiological study of human herpesvirus-8 infection and the risk of multiple myeloma

**DOI:** 10.1054/bjoc.2000.1527

**Published:** 2001-01-01

**Authors:** R Tedeschi, M Kvarnung, P Knekt, T F Schulz, L Szekely, P De Paoli, A Aromaa, L Teppo, J Dillner

**Affiliations:** Microbiology-Immunology and Virology Department, Centro di Riferimento Oncologico-Istituto Nazionale Tumori, Aviano, I-33081, Italy; Laboratory of Tumour Virus Epidemiology, The Microbiology and Tumour Biology Centre, Karolinska Institute, Stockholm, S-17177, Sweden; National Public Health Institute; Social Insurance Institution, Helsinki, Finland; Molecular Virology Group, Dept. of Medical Microbiology and Genitourinary Medicine, the University of Liverpool, UK; Cancer Registry of Finland, Helsinki, Finland

**Keywords:** epidemiology, tumour virology, nested case-control study

## Abstract

Presence of the Human Herpesvirus 8 (HHV8) genome has been reported in the bone marrow of multiple myeloma (MM) patients. So far, serological studies of HHV8 and MM have been inconsistent but have not included prospective epidemiological studies. We evaluated whether HHV8 infection is associated with increased risk for MM in a prospective population-based study of 39 000 Finnish subjects who donated serum samples in the period 1968–72. Serum samples from 47 subjects who developed MM during a 23-year follow-up and 224 age, area of residence and sex-matched subjects who remained healthy over a similar follow-up period were evaluated for HHV8 antibodies at enrolment, as assayed both with an immunofluorescence assay (IFA) for lytic and latent HHV8 antigens and by Western blot (WB) with three recombinant HHV8 proteins (ORFs 65, 73 and K8.1A). HHV8 seropositivity for at least one HHV8 protein on WB was found in 7% of the Finnish population and was not associated with the risk of developing MM (Relative Risk (RR) = 0.89, Confidence Interval (CI): 0.25–3.25). HHV8 seropositivity for lytic and latent antigens in the IFA was found in 16% and 0.4% of the Finnish population and tended to associate with risk of MM (RR = 2.02, CI: 0.94–4.33 and RR = 10.00, CI: 0.91–110.29, respectively). In conclusion, no statistically significant evidence for an association between HHV8 infection and the risk of future MM was found. © 2001 Cancer Research Campaign http://www.bjcancer.com


*British Journal of Cancer* (2001) **84**(1), 122–125
© 2001 Cancer Research Campaign
doi: 10.1054/ bjoc.2000.1527, available online at http://www.idealibrary.com on IDEAL®

http://www.bjcancer.com

### A prospective seroepidemiological study of human herpesvirus-8 infection and the risk of multiple myeloma

**R Tedeschi[1,2], M Kvarnung[2], P Knekt[3,4], TF Schulz[5], L Szekely[2], P De Paoli[1], A Aromaa[3,4], L Teppo[6] and J Dillner[2]**

[1]Microbiology-Immunology and Virology Department, Centro di Riferimento Oncologico-Istituto Nazionale Tumori, Aviano, Italy I-33081;
[2]Laboratory of Tumour Virus Epidemiology, The Microbiology and Tumour Biology Centre, Karolinska Institute, Stockholm, Sweden S-17177;
[3]National Public Health Institute and [4]Social Insurance Institution, Helsinki, Finland; [5]Molecular Virology Group, Dept. of Medical Microbiology and Genitourinary Medicine, the University of Liverpool, UK; [6]Cancer Registry of Finland, Helsinki, Finland

**Summary** Presence of the Human Herpesvirus 8 (HHV8) genome has been reported in the bone marrow of multiple myeloma (MM) patients. So far, serological studies of HHV8 and MM have been inconsistent but have not included prospective epidemiological studies. We evaluated whether HHV8 infection is associated with increased risk for MM in a prospective population-based study of 39 000 Finnish subjects who donated serum samples in the period 1968–72. Serum samples from 47 subjects who developed MM during a 23-year follow-up and 224 age, area of residence and sex-matched subjects who remained healthy over a similar follow-up period were evaluated for HHV8 antibodies at enrolment, as assayed both with an immunofluorescence assay (IFA) for lytic and latent HHV8 antigens and by Western blot (WB) with three recombinant HHV8 proteins (ORFs 65, 73 and K8.1A). HHV8 seropositivity for at least one HHV8 protein on WB was found in 7% of the Finnish population and was not associated with the risk of developing MM (Relative Risk (RR) = 0.89, Confidence Interval (CI): 0.25–3.25). HHV8 seropositivity for lytic and latent antigens in the IFA was found in 16% and 0.4% of the Finnish population and tended to associate with risk of MM (RR = 2.02, CI: 0.94–4.33 and RR = 10.00, CI: 0.91–110.29, respectively). In conclusion, no statistically significant evidence for an association between HHV8 infection and the risk of future MM was found. © 2001 Cancer Research Campaign http://www.bjcancer.com

**Keywords:** epidemiology; tumour virology; nested case-control study

Kaposi's sarcoma associated herpesvirus (KSHV), also denoted HHV8, is a γ-herpesvirus closely associated with Kaposi's sarcoma (KS), body-cavity-based lymphoma and multicentric Castleman's disease (Cesarman et al, 1995; Moore and Chang, 1995). An association has been reported also between multiple myeloma (MM) and HHV8 (Rettig et al, 1997), based on presence of HHV8 DNA in dendritic cells in the bone marrow of MM patients, but not of healthy subjects. This finding was confirmed in some reports (Said et al, 1997; Chauhan et al, 1999) but not in others (MacKenzie et al, 1997; Parravicini et al, 1997; Mitterer et al, 1998). HHV8 encodes a homologue of the cytokine IL-6, known to be required for MM pathogenesis (Moore et al, 1996; Burger et al, 1998), and expression of the viral IL-6 has been reported in dendritic cells of MM patients, providing a plausible hypothesis on a mechanism whereby HHV8 might be involved in MM pathogenesis.

Several serologic studies using both immunofluorescence assays (IFA) and ELISA methods have reported a lack of association between HHV8 and MM (Masood et al, 1997; MacKenzie et al, 1997; Parravicini et al, 1997; Bouscary et al, 1998; Perna et al, 1998; Santarelli et al, 1998). Only one study (Gao et al, 1998), using a Western blot assay with recombinant ORF65 and ORF73 antigens, reported an increased seroprevalence for HHV8 in MM patients, with 81% and 52% of reactivity against ORF65 and ORF73 respectively, as compared to 6% and 2% among healthy control subjects. However, none of these studies were prospective in design. Prospective studies can provide information on temporality and can avoid several sources of selection bias. Furthermore, the possibility exists that the hypergammaglobulinemia seen in MM may have affected serological measurements, causing differential misclassification bias.

To evaluate whether HHV8 infection is associated with an increased risk of MM, avoiding the possibility that the presence of the MM disease might affect measurements, we performed a prospective study nested in a cohort of 39 057 healthy individuals that were followed up for 23 years. For comprehensive evaluation, HHV8 infection was scored both using modern Western blot assays based on recombinant major immunogenic HHV8 proteins (ORF65, ORF K8.1A and ORF73) as well as the reference method, monoclonal antibody-enhanced immunofluorescence assay (mIFA) for lytic and latent HHV8 antigens.

*Received 19 June 2000*
*Revised 18 September 2000*
*Accepted 18 September 2000*

*Correspondence to:* J Dillner

### MATERIALS AND METHODS

### Study design

Between 1968 and 1972 the Mobile Clinic of the Social Insurance Institution of Finland performed a health survey on a population-based sample of 20 243 men and 18 814 women resident in most parts of Finland. The survey included a health interview (including marital status and smoking status), some routine physiological measurements (such as body-mass index) and collection of serum sample (Knekt et al, 1988). A registry linkage of the serum bank with the files of the nationwide Finnish Cancer Registry identified 47 myeloma cases during a follow-up to the end of 1991. For each MM patient, 5 control subjects who were free of any cancer at baseline and who remained alive and free of myeloma up to the time of diagnosis of the corresponding case were selected, matched for gender, age and municipality of residence. Due to the way the cohort was enrolled, the matching for municipality of residence also matched for the time of sample collection. Controls closest in age to the corresponding case were selected. In 79% of the case-control sets the maximal difference between the cases and controls was one year; in 9% of sets it was 3 years or more. Altogether, the serum samples of 224 controls were found and included in the study. The patients gave informed consent at enrolment, the registry linkages were approved by the Computer Inspection Agency and the study by the Institutional Review Board.

The serum samples were coded and analysed without knowledge of the identity of the samples. The serological results were sent to the National Public Health Institute in Helsinki, Finland, where the code was broken and the results were statistically analysed.

### *Proteins*

4 antigens were used in the Western blot assays: GST (glutathione-S-transferase), ORF65, ORF73 and ORFK8.1A. GST was used as a negative control, as any antibody response to this protein in the tested sera is not KSHV specific.

### *ORF73*

A 418 nucleotide long fragment (from the C-terminus) from ORF73 was PCR-amplified using a 5′ primer (GAGGATCC-GAATACCGCTATGTACTCAG) and a 3′ primer (CTGAATTCC-TAGGCGGGCCATTTGTACT) in glass capillaries using a Rapid Cycler (Idaho Technologies). Amplified fragments were subcloned into the LNA-GST2TK (or PGEX 2TK) vector (Pharmacia). The bacterial cultures were grown in fresh LB medium (0.2% glucose and 100 μg ml$^{-1}$ AMP) for two hours at 37˚C. Induction of fusion protein was done by adding isopropyl-beta-D-thiogalacto-pyranoside (IPTG) 0.3 mM for 45 minutes at 37˚C. The protein was purified using Glutatione Sepharose.

### *ORF65*

A recombinant ORF65 protein (aa 86–170) was fused to the carboxy-terminal end of mouse dihydrofolate reductase, placed under control of the T7 promoter in the expression vector pQE 42 (Qiagen, Hilden, Germany), which provides a histidine tag at the aminoterminal end of the fusion protein. The protein was purified to homogeneity using affinity chromatography on Ni-NTA resin (Qiagen) (Simpson et al, 1996).

### *ORFK8.1A*

Purified K8.1A protein was generously provided by Dr Bala Chandran, Dept. of Microbiology, Molecular Genetics and Immunology, the University of Kansas Medical Center, Kansas City, Kansas. ORFs K8.1A and K8.1B had been amplified using a 5′ primer (CGGGGATCCATGAGTTCCACACAGATTCGC) and a 3′ primer (ATGGTCTCGAGTTACACTATGTAGGGTTTC). Amplified PCR fragments had been purified and ligated into pGEM-T vector (Promega) and orientation verified by restriction enzyme digestion and sequence analysis. These plasmids had been digested with BamH1 and XhoI and inserted into the GST expression vector pGEX-4T-1 and orientation verified by restriction enzyme digestion. Induction of fusion protein was done by adding IPTG (1 mM) to bacterial cultures grown to an OD600 of 0.2–0.5. The induced GST-ORFK8.1A fusion protein had been purified using Glutatione Sepharose 4B (Pharmacia) (Chandran et al, 1998).

### *Western blot assay*

271 coded serum samples were tested in the WB reactions in a blinded manner against the antigens GST, ORF65, ORF73 and ORFK8.1A. In each batch of analysis, 10 serum samples and 2 positive controls (pools of serum samples) were used: pool 1 consisted of sera from 6 KS-positive and HIV-negative patients; pool 2 consisted of sera from 5 KS-positive and HIV-positive patients.

The protein samples were separated using SDS-PAGE, 10–15% polyacrylamide gradient gels and electrophoretically transferred to nitrocellulose sheets. After the transfer, the nitrocellulose sheets were cut into 12 strips and blocked with 5% non-fat dry milk in PBS (MilkPBS), for 1 h at room temperature (RT). Human sera (1:50 diluted in 2% MilkPBS) were then incubated with the strips for 2 h at RT, shaking. The strips were washed 5 times with 5% non-fat dry milk and finally incubated for 30 minutes with goat anti-human IgG HRP conjugate (Dako), diluted 1/1000 in 2% MilkPBS. After three washes with 2% MilkPBS and several washes with PBS-0.5% Tween20, bound enzyme-labelled antibodies were detected using ECL Western blotting detection reagent (Amersham), according to the manufacturer’s instructions.

### Immunofluorescence assay

IFA was performed as described (Lennette et al, 1996). Briefly, $10^7$ HHV8-carrying BCBL-1 cells or HHV8-negative Ramos cells in 10 ml of medium were induced with 10 ng ml$^{-1}$ TPA (Sigma) and 200 UI ml$^{-1}$ IL-6 (Beckton Dickinson) for 5 days. TPA-induced BCBL-1 and Ramos cells were collected and washed in PBS and smeared on coverslips, air dried and fixed in acetone. Slides were incubated successively in 3 steps of 30 minutes each at 37˚C with the test serum diluted 1:10, with mouse monoclonal anti-human IgG 1, 2, 3 (ATCC HP6058) and then with FITC-labelled anti-mouse antibodies (1:80 diluted; Sigma) and Evans’ blue counterstain. Two independent observers scored lytic and latent seropositivities. Specific granular punctate nuclear fluorescence with the majority (>80%) and bright cytoplasmic fluorescence with about 30% of the BCBL-1 cells were considered as recognizing latent and lytic HHV8 antigens, respectively (Lennette et al, 1996). Sera were tested with a starting dilution of 1:10 and then titred with 4-fold serum dilutions. Sera that showed non-specific fluorescence against the Ramos cell negative control were scored as not specifically reactive.

### Statistical analysis

The laboratory results were transmitted to the Finnish Social Insurance Institution where the code was broken and the relative risk (RR) of developing MM given HHV8 seropositivity was estimated using conditional logistic regression.

### RESULTS

### Validation of the sensitivities of the serologic tests

The sensitivities of the assays were evaluated using sera from 45 AIDS patients and 10 HIV-negative patients attending the Division of AIDS & Medical Oncology at the Centro di Riferimento

*© 2001 Cancer Research Campaign* 

**Table 1** Reactivity of human Kaposi's sarcoma sera with HHV8 ORFs K8.1A, 65 and 73 recombinant proteins on Western blot and IFA assays

| | N° | WB ORFs K8.1A/65/73 positive | WB ORFK8.1A positive | WB ORF65 positive | WB ORF73 positive | IFA Latent positive | IFA Lytic positive |
|---|---|---|---|---|---|---|---|
| KS HIV+ Italy | 45 | 37 (82%) | 26 (58%) | 29 (64%) | 12 (27%) | 21 (47%) | 42 (93%) |
| KS HIV− Italy | 10 | 10 (100%) | 7 (70%) | 10 (100%) | 5 (50%) | 7 (70%) | 9 (90%) |
| KS HIV− Africa | 17 | 16 (94%) | 15 (88%) | 9 (53%) | 7 (41%) | 16 (94%) | 16 (94%) |
| Total | 72 | 63 (88%) | 48 (67%) | 48 (67%) | 24 (33%) | 44 (61%) | 67 (93%) |

Oncologico in Aviano with the diagnosis of KS and 17 serum samples from HIV-negative patients with KS collected in 1973–1974 in Kenya (Table 1). The sensitivities ranged from 33% for ORF73 Western blot to 93% for lytic immunofluorescence, which is comparable to what has been reported in previous studies.

### Comparison of reactivities of sera with HHV8 infected BCBL-1 cells on mIFA and with the recombinant HHV8 proteins on Western Blot Assay

Altogether 20/271 (7.4%) serum samples from the prospective study were reactive with at least one HHV8 recombinant protein in WB.

In immunofluorescence, 18% (46 of 271) of the serum samples were positive only for the lytic IFA antibodies and 1% (3 of 271) were positive for both the latent and lytic IFA antibodies. The titres ranged from 1:10 to ≥1:640.

The level of agreement among mIFA (lytic or latent) and WB (considering the reactivity against at least one antigen) was not very high (Odds Ratio = 12.36, CI: 5.36–28.08) (Table 2). Immunofluorescence identified more samples as positive (49 vs 20); 13 samples were concordantly positive and 209 concordantly negative, on both of these tests.

### Risk of multiple myeloma among HHV8 seropositive subjects

Western blot seropositivity had no association with subsequent MM occurrence (RR: 0.89, CI: 0.25–3.25). The point estimates of the RR for the different antigens varied from 0.37 for ORF65 to 3.18 for ORFK8.1A, but none of the RRs were significantly different from unity. Only 7 samples were concomitantly positive for >1 antigen in Western blot. Positivity for multiple antigens was not associated with MM (RR: 0.96; CI: 0.10–8.82). The estimated relative risk for MM was increased both for the lytic and for the latent immunofluorescence (OR: 2.0 and 10.0, respectively), albeit this was not statistically significant (Table 3).

The immunofluorescence positive serum samples and a random subset of 16 negative serum samples were subjected to repeat analysis. The negative samples again tested negative. The positive samples tested positive again, although only in 32 cases was the titre the same. 11 serum samples had a lower titre by one dilution step and four serum samples a higher titre by one dilution step. The Western blot positive sera were also retested with confirmatory results.

**Table 2** Comparison between IFA and Western blot HHV8 reactivity

| | WB ORFK8.1A | WB ORF65 | WB ORF73 | IFA LYT | IFA LAT |
|---|---|---|---|---|---|
| WB ORFK8.1A | 1.0 | – | – | – | – |
| WB ORF65 | 0.34* | 1.0 | – | – | – |
| WB ORF73 | 0.15* | 0.11* | 1.0 | – | – |
| IFA LYT | 0.29* | 0.34* | 0.10 | 1.0 | – |
| IFA LAT | 0.18* | 0.14* | 0.28* | 0.22* | 1.0 |

Spearman correlation coefficients; *: $P < 0.05$.

**Table 3** HHV8 seropositivity and risk of multiple myeloma

| | | Multiple myeloma on follow-up | Healthy on follow-up | RR | 95% CI |
|---|---|---|---|---|---|
| IFA lytic | No | 34 | 188 | Ref. | |
| | Yes | 13 | 36 | 2.02 | 0.94–4.33 |
| IFA latent | No | 45 | 223 | Ref. | |
| | Yes | 2 | 1 | 10.00 | 0.91–110.29 |
| WB: any one of ORF65, ORF73 or ORFK8.1A | No | 44 | 208 | Ref. | |
| | Yes | 3 | 16 | 0.89 | 0.25–3.25 |
| WB ORF65 | No | 46 | 212 | Ref. | |
| | Yes | 1 | 12 | 0.37 | 0.05–2.99 |
| WB ORF73 | No | 47 | 220 | Ref. | |
| | Yes | 0 | 4 | 0.00 | 0.00 |
| WB ORF K 8.1A | No | 44 | 219 | Ref. | |
| | Yes | 3 | 5 | 3.18 | 0.70–14.47 |

### DISCUSSION

We aimed to evaluate whether HHV8 infection was associated with an increased risk for multiple myeloma. Since the initial report that hypothesized that KSHV causes MM through infection of bone marrow stromal dendritic cells and possible paracrine effects of viral IL-6 (Rettig et al, 1997), several studies have addressed the presence of HHV8 DNA in bone marrow cells and

*© 2001 Cancer Research Campaign*

HHV8 seroprevalences among MM patients, with mixed results. The most likely reasons for mixed results are different reliabilities of the serological methods used and selection biases in studies performed without a formal study base definition.

Our serologic study evaluated whether HHV8 infection is associated with increased risk for multiple myeloma in a defined cohort of 39 057 subjects that were followed up for 23 years. The prospective study design avoided the possibility that MM disease itself might affect serological measurements. For example, the markedly decreased general humoral response in this disease might impair HHV8 antibody response. A 20-year follow-up may be required to evaluate the impact of infection on emergence of virus-associated cancers (zur Hausen, 1999).

Our serological evaluation used several of the different HHV8 antibody detection methods: Western blot assay based on 3 different recombinant proteins as well as immunofluorescence. The different rates of HHV8 seroprevalence using different methods is likely to reflect differences in assay sensitivities, specificities and/or reproducibilities (Rabkin et al, 1997). Repeat testing of the same samples at a later time point did not find any noteworthy differences in the reproducibility of the different tests used. The positive control serum panel of patients with KS was used for within-study validation of sensitivity. As it is not possible to define serum panels of definitely unexposed persons, the population-based healthy controls of the study could be used for a crude estimation of specificity. It is interesting to note that if the validity of the assays is ranked by odds ratios for association with KS in comparison with healthy controls, the different serological assays rank in the order latent IF, ORF K8.1 WB, lytic IF, ORF 65 WB and ORF73 WB, suggesting that the validity of the assay correlates with the point estimate of the multiple myeloma risk (cf.Table 3).

In conclusion, HHV8 seropositive persons were not found to be at increased risk of MM. Since the statistical power of our study was limited, further studies may be warranted to evaluate whether HHV8 infection is associated with MM.

### ACKNOWLEDGEMENTS

Grant Support: RT is supported by the Foundation Blanceflor Ludovisi, nee' Bildt. TFS was supported by the NHS Biomedical Research Fund, RDO/22/9.

### REFERENCES

Bouscary D, Dupin N, Fichelson S, Grandadam M, Fontenay-Roupie M, Marcelin AG, Blanche P, Picard F, Freyssinier JM, Ravaud P, Dreyfus F and Calvez V (1998) Lack of evidence of an association between HHV-8 and Multiple Myeloma. *Leukemia* **12**: 1840–1841

Burger R, Neipel F, Fleckenstein B, Savino R, Ciliberto G, Calden JR and Gramatzki M (1998) Human Herpesvirus type 8 interleukin-6 homologue is functionally active on human myeloma cells. *Blood* **91**: 1858–1863

Cesarman E, Chang Y, Moore PS, Said JW and Knowles DM (1995) Kaposi's sarcoma associated herpesvirus-like DNA sequences in AIDS-related body cavity based lymphomas. *New Engl J Med* **332**: 1186–1191

Chandran B, Bloomer C, Chan SR, Zhu L, Goldstein E and Horvat R (1998) Human Herpesvirus-8 ORF K8.1 gene encodes immunogenic glycoproteins generated by spliced transcripts. *Virology* **249**: 140–149

Chauhan D, Bharti A, Raje N, Gustafson E, Pinkus JL, Teoh G, Hideshima T, Treon SP, Fingeroth JD and Anderson KC (1999) Detection of Kaposi's sarcoma herpesvirus DNA sequences in multiple myeloma bone marrow stromal cells. *Blood* **93**(5): 1482–1486

Gao SJ, Alsina M, Deng JH, Harrison CR, Montalvo EA, Leach CT, Roodman GD and Jenson HB (1998) Antibodies to Kaposi's sarcoma-associated Herpesvirus (Human Herpesvirus 8) in patients with Multiple Myeloma. *J Infect Dis* **178**: 846–849

Knekt P, Aromaa A, Maatela J, Aaran RK, Nikkari T, Hakama M, Hakulinen T, Peto R, Saxen E and Teppo L (1988) Serum vitamin E and risk of cancer among Finnish men during a 10 year follow-up. *Am J Epidemiol* **127**(1): 28–41

Lennette ET, Blackbourn DJ and Levy JA (1996) Antibodies to Human herpesvirus type 8 in the general population and in Kaposi's sarcoma patients. *Lancet* **348**: 858–861

MacKenzie J, Sheldon J, Morgan G, Cook G, Schulz TF and Jarrett RF (1997) HHV-8 and multiple myeloma in the UK. *Lancet* **350**: 1144–1145

Masood R, Zheng A, Arora N, Chatlynne L, Handy M, Whitman J, Kaplan M, Dosik M, Ablashi DV and Gill PS (1997) Kaposi's sarcoma associated Herpesvirus infection and multiple myelomas. *Science* **278**: 1970–1971

Mitterer M, Mair W, Gatti D, Sheldon J, Vachula M, Coser P and Schulz TF (1998) Dendritic cells derived from the bone marrow and CD34+ selected blood progenitor cells of myeloma patients, cultured in serum-free media, do not contain the Kaposi's sarcoma herpesvirus genome. *Br J Haematol* **102**: 1338–1342

Moore PS and Chang Y (1995) Detection of herpesvirus-like DNA sequences in Kaposi's sarcoma patients with and without HIV infection. *New Engl J Med* **332**: 1181–1185

Moore PS, Boshoff C, Weiss RA and Chang Y (1996) Molecular mimicry of human cytokine and cytokine response pathway genes by KSHV. *Science* **274**: 1739–1744

Parravicini C, Lauri E, Baldini L, Neri A, Poli F, Sirchia G, Morono M, Galli M and Corbellino M (1997) Kaposi's sarcoma-associated Herpesvirus infection and Multiple Myeloma. *Science* **278**: 1969–1973

Perna AM, Viviano E, Iannitto E, Marcenò R and Romano N (1998) No association between Human Herpesvirus type 8 infection and Multiple Myeloma. *J Natl Cancer Inst* **90**(13): 1013–1014

Rabkin CS, Schulz TF, Whitby D, Lennette ET, Magpantay LI, Chatlynne L and Biggar R (1997) Interassay correlation of Human Herpesvirus 8 serologic tests. *J Infect Dis* **178**: 304–309

Rettig MB, Ma HJ, Vescio RA, Pold M, Schiller G, Belson D, Savage A, Nishikubo C, Wu C, Fraser J and Said JW (1997) Kaposi's sarcoma associated Herpesvirus infection of bone marrow dendritic cells from multiple myeloma. *Science* **276**: 1851–1854

Said JW, Rettig MR, Heppner K, Vescio RA, Schiller G, Ma HJ, Belson D, Savage A, Shintaku IP, Koeffler HP, Asou H, Pinkus G, Pinkus J, Schrage M, Green E and Berenson JR (1997) Localization of Kaposi's sarcoma-associated herpesvirus in bone marrow biopsy samples from patients with multiple myeloma. *Blood* **90**: 4278–4282

Santarelli R, Angeloni A, Farina A, Gonnella R, Gentile G, Martino P, Petrucci MT, Mandelli F, Frati L and Faggioni A (1998) Lack of serologic association between Human Herpesvirus-8 infection and multiple myeloma and monoclonal gammopathies of undetermined significance. *J Natl Cancer Inst* **90** (10): 781–782

Simpson GR, Schulz TF, Whitby D, Cook PM, Boshoff C, Rainbow L, Howard MR, Gao SJ, Bohenzky RA, Simmonds P, Lee C, de Ruiter A, Hatzakis A, Tedder RS, Weller IVD, Weiss R and Moore P (1996) Prevalence of Kaposis's sarcoma associated Herpesvirus infection measured by antibodies to recombinant capsid protein and latent immunofluorescent antigen. *Lancet* **349**: 1133–1138

zur Hausen H (1999) Viruses in human cancers. *Eur J Cancer* **35**: 1174–1181

© 2001 Cancer Research Campaign 
